# Highly conducting Laser-Induced Graphene-Ag nanoparticle composite as an effective supercapacitor electrode with anti-fungal properties

**DOI:** 10.1038/s41598-024-79382-3

**Published:** 2024-11-13

**Authors:** Abhishek Prakash, Sowmya R. Holla, Subbalaxmi Selvaraj, Ramakrishna Nayak, Shounak De, Mohammad Saquib, M. Selvakumar

**Affiliations:** 1 Department of Electronics & Communication Engineering, Manipal Institute of Technology, Manipal Academy of Higher Education (MAHE), Manipal, 576104 India; 2 Department of Chemistry, Manipal Institute of Technology, Manipal Academy of Higher Education (MAHE), Manipal, 576104 India; 3 Department of Biotechnology, Manipal Institute of Technology, Manipal Academy of Higher Education (MAHE), Manipal, 576104 India; 4 Department of Humanities and Management, Manipal Institute of Technology, Manipal Academy of Higher Education, Manipal, Karnataka 576104 India

**Keywords:** Green-synthesis, Silver Nanoparticles, Laser-Induced Graphene, Microsupercapacitor, Antifungal agent, Biotechnology, Energy science and technology, Materials science

## Abstract

This study presents a simple and an environmentally friendly approach to make Laser-Induced Graphene (LIG) based supercapacitor electrodes anchored with abundant Silver Nanoparticles (AgNPs). LIG, was synthesized using a CO_2_ laser writing technique on polyimide substrate. The LIG-Ag composite was prepared using two techniques, drop-coating, and screen-printing. Ag nanoparticles prepared using the plant extract of *Swietenia Macrophylla* was utilized to drop-coat AgNP on LIG substrate. Screen-printing was done by using a commercial Ag-ink and a suitable mesh. The supercapacitor made from screen-printed electrodes and supercapacitor made form drop-coated electrodes showed a high specific capacitance of 118 mF/cm^2^, 38 mF/cm^2^, and a high energy density of 2.42 mWh/cm^2^, 0.05 mWh/cm^2^ respectively. The screen-printed composite of LIG and AgNP was further studied for its anti-fungal properties and proved to be effective against *Candida sp*.

## Introduction

Our goals for a sustainable future and a circular economy requires the development of green energy technologies such as batteries and supercapacitors for energy generation and storage^1–3^. Supercapacitors have been highly sought after in recent times owing to their high-power density and energy density comparable to that of batteries ^4–8^. Thus, their applications are immense, spanning from electromobility to wearable devices. In recent times, flexibility has become an important characteristic for energy storage devices for nanoelectronics. In this respect, Laser-Induced Graphene has become highly popular owing to its cost effectiveness of preparation, repeatability, and scalability^9–12^. Graphene, an EDLC material has diverse properties such as high surface area, porosity, and high thermal stability^13–15^. Additionally, EDLCs have high cycling stability and power density. However, their energy density and capacitance are limited ^4^. Hybrid conformation of an EDLC material with a pseudocapacitive material has proven to be greatly effective to overcome this shortcoming^16^. Various binder-free electrodes have been prepared and reported. For example, Clerici and workers decorated Laser-Induced Graphene with MoS_2_ nanoparticles by coating MoS_2_ on the polyimide substrate followed by laser-ablation ^17^. Doping with Phosphorus, Nitrogen, Boron, and co-doping has also been reported, resulting in excellent micro supercapacitors ^14,18,19^. Seyed et al. reported a silver nanowire containing Laser-Induced Graphene electrode which was further used to construct a symmetric flexible supercapacitor ^20^.

Green techniques such as utilization of plant extract for nanoparticle synthesis has paved ways to make active nanoparticle species with natural capping agents. These species have been widely studied for antibacterial, antifungal, and anticancer activities. For example, Mohammed et al*.* synthesized Ag nanoparticles using *Conocarpus Lancifolius* plant extract and studied them for their antimicrobial and anticancer activities ^21^. Gunasekaran et al*.* synthesized Au nanoparticles using *Jatropha Integerrima* Jacq. flower extract and studied them for their antibacterial activity ^22^. Bassant et al. synthesized Zinc Oxide nanoparticles using Sea Lavender extract and tested for their anti-cancer, anti-microbial, and antioxidant activities ^23^.

*Candida sp.* are species of pathogens that causes fungal infections, which greatly affects the skin, gastrointestinal tract, and is a major cause for systemic infection. There are as many as 400,000 systemic fungal infections that caused by *Candida sp*. ^24,25^. About 70% of fungal infections worldwide are caused by *Candida albicans*, often responsible for systemic and mucosal infections^26^. In the past few decades, it has been the major cause of infections that are potentially fatal. The mortality rate is close to 40% despite treatment, even with hospital conditions ^27^. Graphene has also been known to exhibit antifungal activity. However, its antifungal activity is found to be a lot lower compared to other carbon materials such as CNTs and MWNTs. This is attributed to weaker Van Der Waals forces and lower aggregation resulting in lack of contact between the spores and Graphene ^28,29^. This can be improved greatly by embedding nanoparticles onto Graphene ^30–32^. This would lead to increase in interlayer spacing and decrease in Van Der Waal forces due to potential intercalation of nanoparticles within the Graphene layers. However, this would be overshadowed by the enhanced activity of the Graphene-nanoparticle composite.

Through this work, for the first time we developed a silver nanoparticle anchored composite (AgNP@LIG) which proved to be highly effective against pathogenic *Candida sp.* and highly effective electrode for a microsupercapacitor. The Ag nanoparticles were synthesized by reduction of AgNO_3_ using the plant extract of *Swietenia Macrophylla* Bark and were coated on LIG using both screen-printing and drop coating. A symmetric supercapacitor was created using the latter. For comparative study, Ag nanoparticles were screen-printed on LIG using a commercial ink, and a symmetric supercapacitor was made from this electrode. Lastly, the screen-printed electrode was studied for its anti-fungal properties and turned out positive against *Candida sp.* This study showed that LIG-Ag nanoparticle composite demonstrates high antifungal activity and unlocks high supercapacitor performance as well.

## Materials and methods

### Synthesis of AgNP

The nanoparticles were prepared by reducing AgNO_3_ to Ag using a Green Method using the powdered version of *Swietenia Macrophylla* Bark. 3 g of the sample was dissolved in 60 mL DI water followed by rigorous shaking in centrifuge tubes for 48 h at 180 rpm and 32⁰ C. The solutions were then centrifuged at 4⁰ C at 10,000 rpm for 15 min. The supernatant obtained was thus separated from the pellet and transferred to another centrifuge tube. 2 mM of AgNO_3_ was prepared. AgNO_3_ and the supernatant were mixed at a 1:2 ratio followed by a 48-h incubation period. The mixture was then transferred to a petri dish and placed in a hot air oven until the solution entirely dried up. The petri dish was scraped using a spatula to obtain the nanoparticles.

### Preparation of LIG

LIG was prepared by laser ablation of Polyimide Sheet in an open atmosphere using a commercial 100 W CO_2_ infrared laser engraver. PI sheet was thus turned into a porous square 10 × 10 mm graphene sheet. The optimal values found to be the following according to Table 1.

**Table 1:** Laser parameters.

Mode	Resolution	Scanning Speed	Laser Power
Raster	400 dpi	180 mm/s	44 W

### Preparation of LIG/AgNP composite

The composite was prepared using 2 techniques: drop-coating and screen-printing. For the former, a certain amount of AgNP was dispersed in Ethanol thoroughly and then drop casted onto LIG, followed by drying at 80 degrees Celsius. For preparing the screen-printed LIG electrode, 0.1 g of AgNP was taken and mixed with a few drops of a homogenous mixture of Diacetone Alcohol and Carboxyl Acetate Propionate and mixed thoroughly. Finally using a mesh, the screen-printing was completed. A screen-printed electrode using commercially available Ag ink was also created using the same mesh. The electrodes were studied for their sheet resistance using the four-probe instrument and the Van der Pauw method. The values have been tabulated in Table 2. The composite comprises around 0.012 g of Ag nanoparticles deposited on a 10 mm × 10 mm Laser-Induced Graphene created using laser-ablation on a Polyimide Sheet.

**Table 2:** Electrode composition and conductivity study.

Electrode Name	Composition	Sheet resistance (Ω)	Conductivity (S cm^-1^)
E_1_	AgNP prepared using green method and drop coated on LIG	37.10	12.2
E_2_	AgNP prepared using green method and screen-printed on LIG	28.25	16.04
E_3_	Commercially available Ag ink screen-printed on LIG	3.00	151.09

### Preparation of gel-polymer electrolyte

**Fig. 1:**
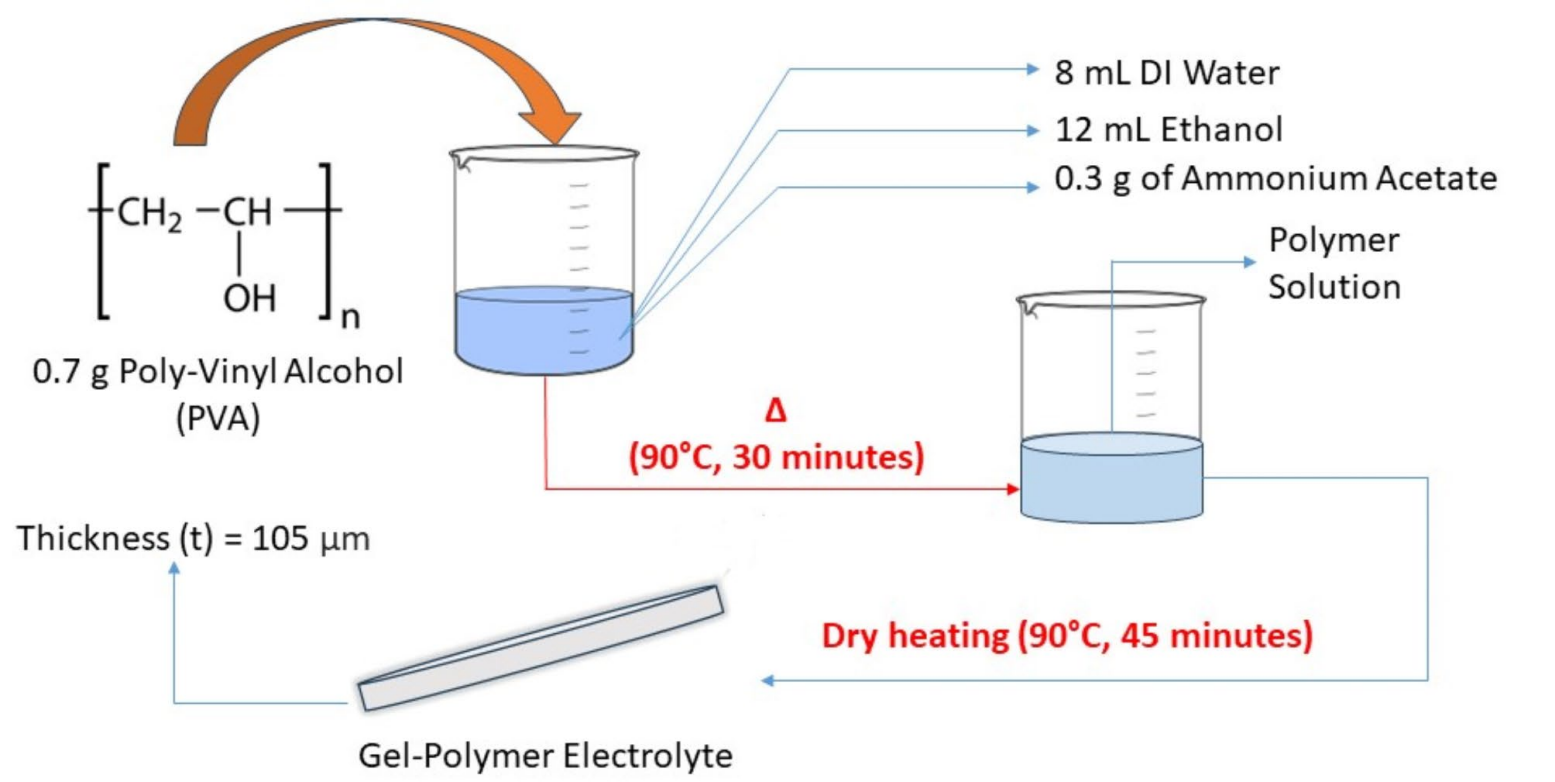
Gel-electrolyte film preparation.

The gel electrolyte was prepared using solution casting method (Fig. 1). The electrolyte solution was prepared by dissolving 0.7 g of Polyvinyl Alcohol (PVA) in a mixture of 8 mL deionized water, 12 mL ethanol, and 0.3 g of ammonium acetate (NH_4_CH_3_CO_2_). The dissolution process involved using a magnetic stirrer and a water bath set at 400 rpm and 90 degrees Celsius, respectively, until a clear solution was obtained. The mixture was then poured into a petri dish and placed in an oven at 90 degrees Celsius, resulting in the formation of a thin, transparent film.

### Supercapacitor fabrication

**Fig. 2:**
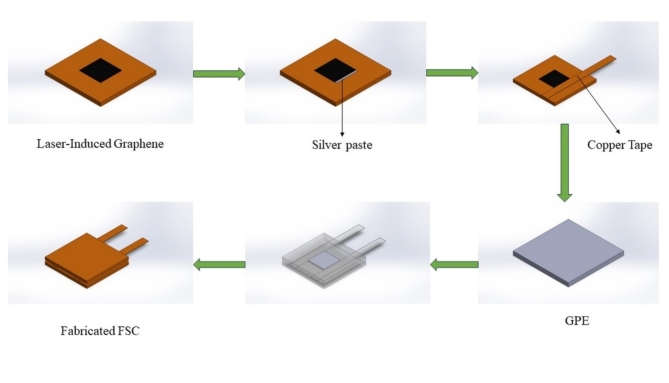
Supercapacitor Fabrication.

A pair of E_1_ and E_3_ electrodes were prepared to make 2 symmetric supercapacitors (Table 3) to compare screen-printing and drop coating methods of composite preparation. The gel polymer electrolyte was sandwiched between the 2 symmetric electrodes as shown in Fig. 2.

**Table 3:** Supercapacitor (SC) Composition.

SC Name	Composition
I	E_1_ | GPE | E_1_
II	E_3_ | GPE | E_3_

### Antifungal studies

The antifungal activity of E_2_ was tested against *Candida sp*. This strain was obtained from the Institute of Microbial Technology, Chandigarh, India. The strain was pre-cultured at 32 °C and 180 rpm for an entire night in nutrient broth medium. Subsequently, the antifungal activity of the composite was assessed using the agar well diffusion method, as previously mentioned ^33,34^. The 0.1 mL of overnight grown strain were spread plated on sterilized and solidified nutrient agar plates. The disc of diameter 0.25 cm of the electrode was punched and soaked in the *Candida sp.* culture and thereafter placed on the aforementioned agar plate. The plates were then incubated at for 24 h at 37⁰ C. The antifungal activity was quantified by measuring the clear zone formation around the disc using a Vernier calliper. The sterilized filter paper disc soaked with DI water of diameter 0.25 cm was used as positive control.

**Fig. 3:**
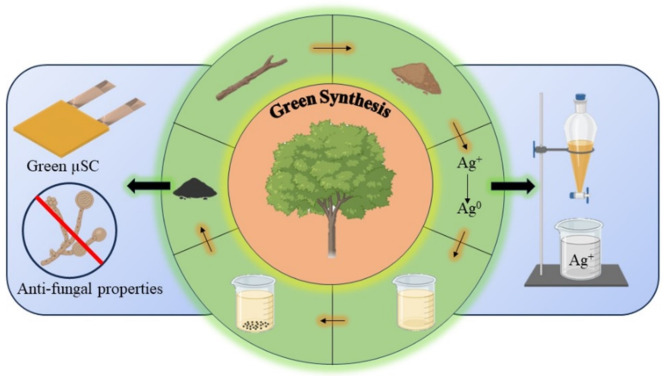
Summary of the methodology.

The entire process, objectives, and deliverables are elucidated as shown in in Fig. 3. Powdered bark has been utilized to realize the conversion of AgNO_3_ to Ag nanoparticles followed by its application for anti-fungal studies and microsupercapacitors.

## Results and discussion

### Electrode characterization

#### SEM of LIG

SEM (Scanning Electron Microscopy) analysis was performed to study the morphology of Laser-Induced Graphene (LIG).

**Fig. 4:**
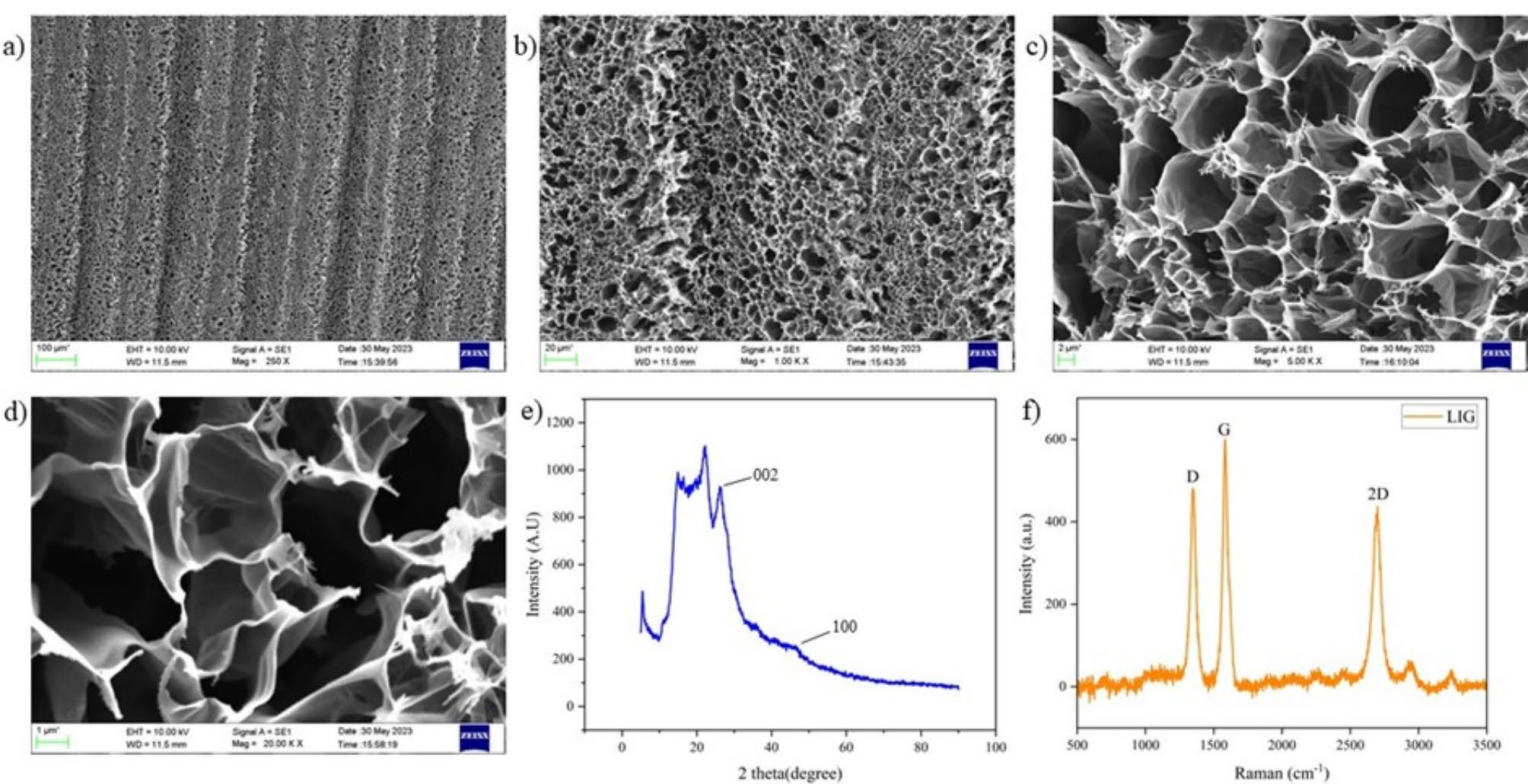
(**a**)–(**d**) SEM of LIG (**e**) XRD of LIG (**f**) Raman Spectroscopy of LIG.

The SEM analysis at various magnifications of the resulting Laser-Induced Graphene (LIG) as shown in Fig. 4(a)–(d), revealed a highly ordered, porous and interconnected network structure indicating successful LIG formation. This is due to the photothermal effect due to high heat generated from laser scribing which induces internal atomic excitation and generates carbonized steam. The SEM images showcase a foam-like appearance which can be attributed to liberation of gases due to laser ablation ^35^.

#### SEM of Screen-printed LIG-Ag composite (E_3_)

**Fig. 5:**
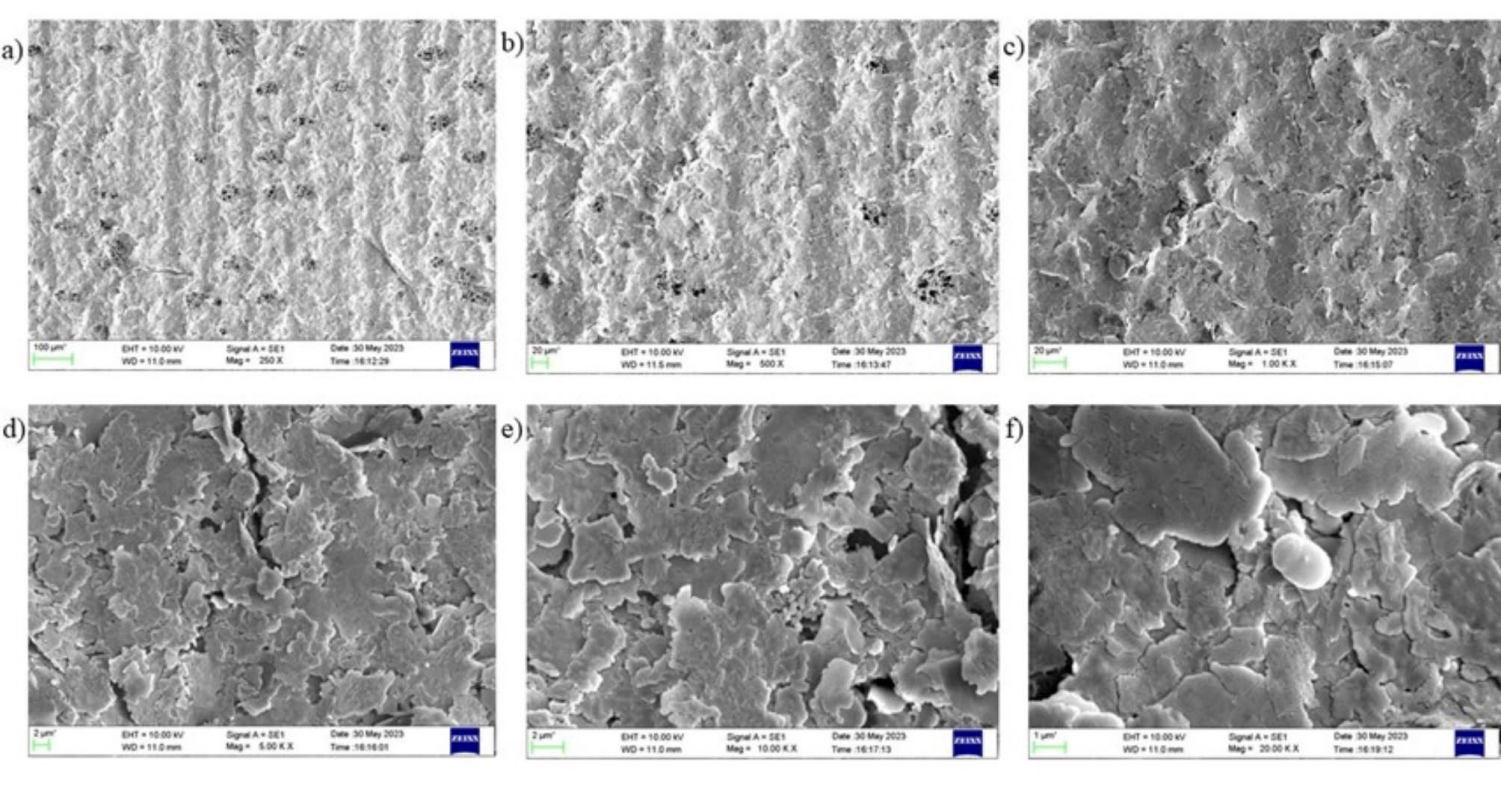
SEM of Screen-printed Electrode E_3_.

The morphology of E_3_ was observed using SEM as well as shown in Fig. 5(a)–(f). The images show a highly aggregated flake-like morphology.

#### XRD of LIG

The X-ray diffraction (XRD) pattern of Laser-Induced Graphene (LIG) exhibits a prominent peak at 2θ = 26.0 degrees as shown in Fig. 4e, indicating the presence of highly crystalline graphene structure. The peak corresponds to the (002) crystal plane, and the interlayer spacing (I_c_) was found to be 3.4 Å. The XRD graph reveals an additional peak at 2θ = 43.0 degrees, corresponding to the (100) reflections ^9^. The additional peaks correspond to the Polyimide sheet which was ablated to obtain LIG ^36^.

#### Raman Spectroscopy of LIG

The Raman spectrum of Laser Induced Graphene (LIG) obtained using Renishaw Raman microscope (wavelength = 432 nm) exhibited three prominent peaks (Fig. 4f). The first is the D peak, observed at approximately 1,350 cm^-1^, which arises from defects or bent sp2-carbon bonds present in the material. The second is the G peak, occurring at around 1,580 cm^-1^, which corresponds to the first order allowed vibration of the carbon atoms in the hexagonal lattice. The third peak is the 2D peak, detected at approximately 2,700 cm^-1^, which originates from second-order zone-boundary phonons. The G and 2D peaks also correspond to number of layers present in Laser-Induced Graphene. The D and G peak intensity ratio (I_D_/I_G_) is a good metric to analyse the degree of disorder within the graphitic lattice. In this case, the I_D_/I_G_ ratio is 0.84 confirming that the LIG produced is of good quality ^37^.

### AgNP characterization

#### FTIR Results

**Fig. 6:**
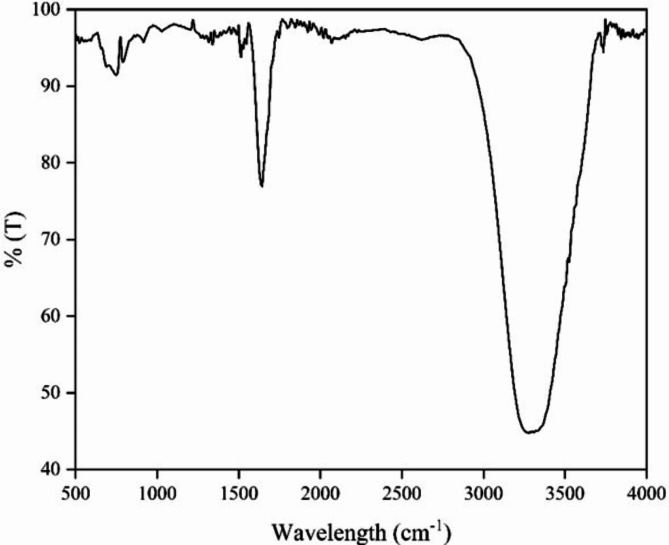
FTIR of reaction mixture of plant extract and AgNO_3_ (2:1).

FTIR study was conducted on the reaction mixture (Supernatant + AgNO_3_) produced right after 48 h incubation (Fig. 6). The 3 transmission peaks at the functional peak region 3734.76 cm^-1^, 3523.71 cm^-1^, 3309.80 cm^-1^ is due to O–H stretching of alcoholic and phenolic compounds. The transmission peak at 1642.78 cm^-1^ can be attributed to C=O stretching, indicating the presence of an ester. The peaks corresponding to 1528.7 cm^-1^, 1522.59 cm^-1^, and 1514.44 cm^-1^ are due to C=C stretching of the Alkenyl or Aromatic groups. The presence of C-O group of the Aliphatic compound can be deduced from the peaks at wavelengths 790.02 cm^-1^, 748.66 cm^-1^, 690.20 cm^-1^. The presence of hydroxyl, ester, and carboxyl groups in the presence of AgNO_3_ are likely to get converted to acids. This is indicative of the reduction of Ag^+^ to Ag and thus indicates the presence of Ag nanoparticles. Presence of various functional groups helps in the reduction and capping of AgNP ^38^.

#### UV Results

**Fig. 7:**
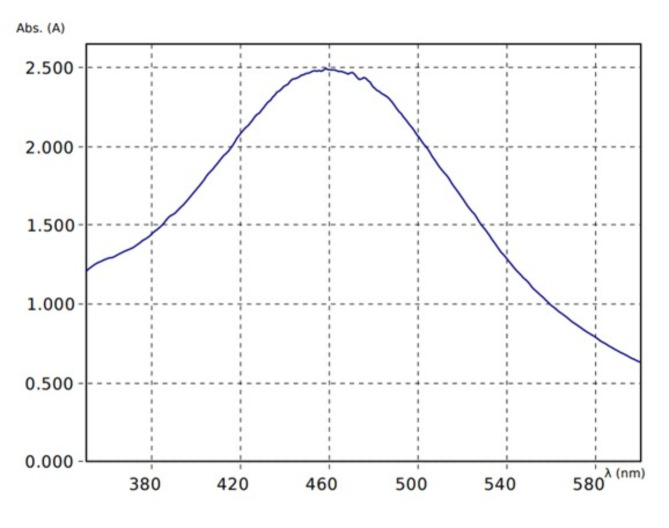
UV Spectroscopy of reaction mixture of plant extract and AgNO_3_ (2:1).

To further detect the presence of Ag nanoparticles, UV spectroscopy of the reaction mixture of the supernatant and AgNO_3_ was conducted. A characteristic peak at 460 nm wavelength (Fig. 7) was obtained owing to the excitation of Surface Plasmon Resonance (SPR) vibrations of silver nanoparticles synthesized in the reaction medium ^39^.

#### SEM and SEM EDX of AgNP

**Fig. 8:**
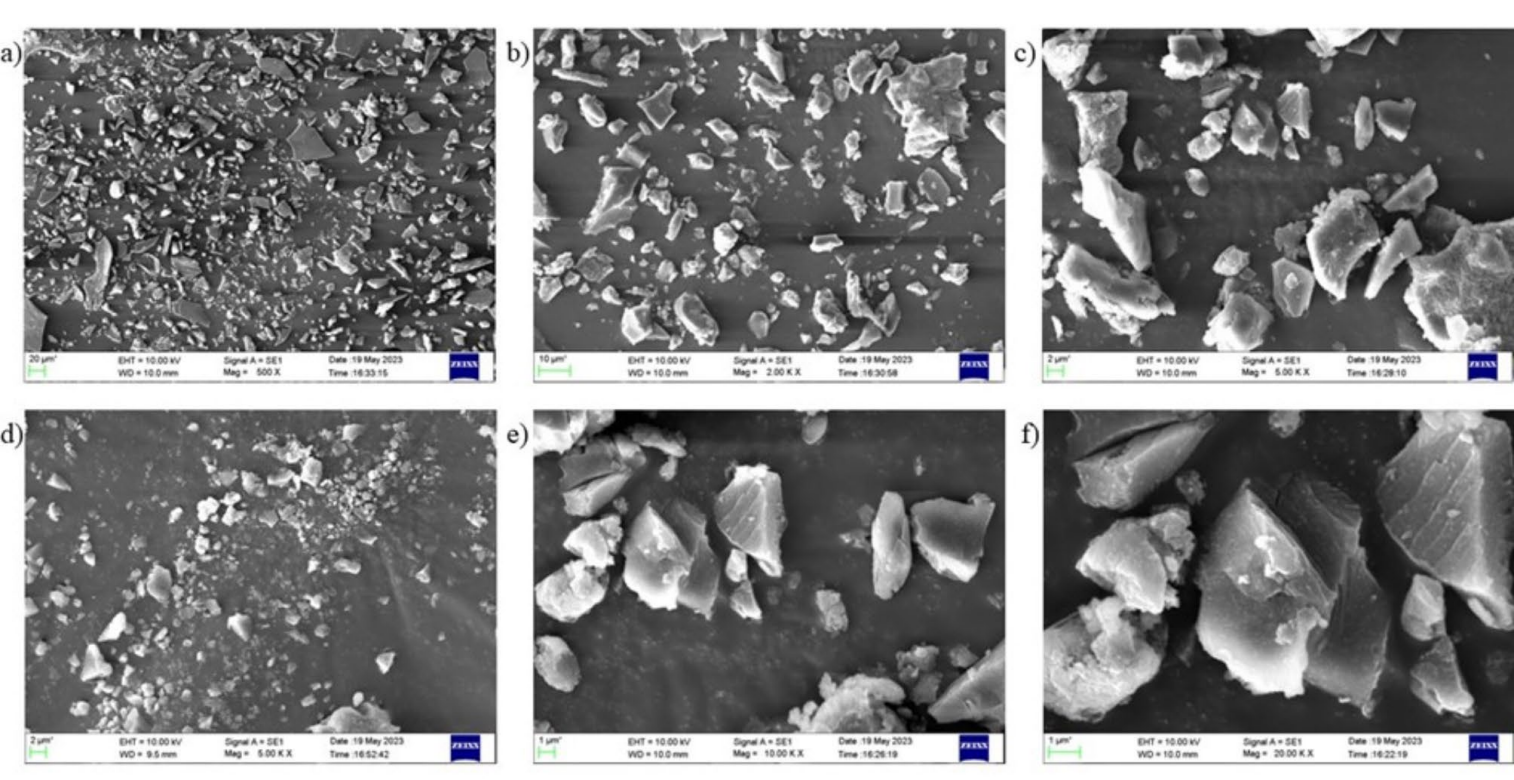
SEM images of Ag nanoparticles.

**Fig. 9:**
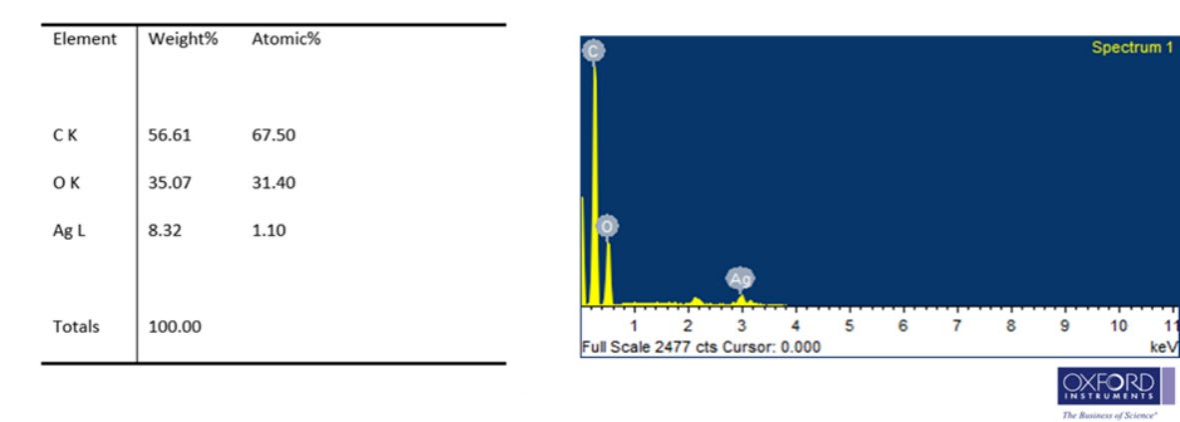
SEM–EDX of Ag nanoparticles.

SEM analysis of AgNP obtained after drying the reaction mixture is shown in Fig. 8(a)-(f). The presence of AgNP in the powder was analyzed by SEM equipped with energy dispersive X-ray spectroscopy (Fig. 9). A strong signal of Ag atoms was visible in the EDX spectra at a wavelength of ~ 2.133 keV. In EDX spectra, the K, C and O peaks are highly evident and significant. The observed peaks for K, C, and O may arise from various sources, including organic residues, or insoluble biproducts.

### Electrochemical studies of supercapacitor

Electrochemical characterization of the supercapacitor was carried out for the 2 symmetric supercapacitors using CV, GCD, and EIS techniques using SP-150, Bio-Logic electrochemical workstation. The following equations were utilized to calculate the specific capacitance from the CV curves and GCD curves respectively:

1 $${C}_{sp}=\frac{\int IdV}{kA\Delta V}$$

2 $${C}_{sp}=\frac{I\Delta t}{\Delta V}$$

where, the integral refers to the area under the CV curve, *A* refers to the area of the active material, *k* refers to the scan-rate used for CV, *Δt* refers to the discharge-time after IR drop, *ΔV* refers to the discharge potential window after the voltage drop ^40^.

#### Cyclic voltammetry

**Fig. 10:**
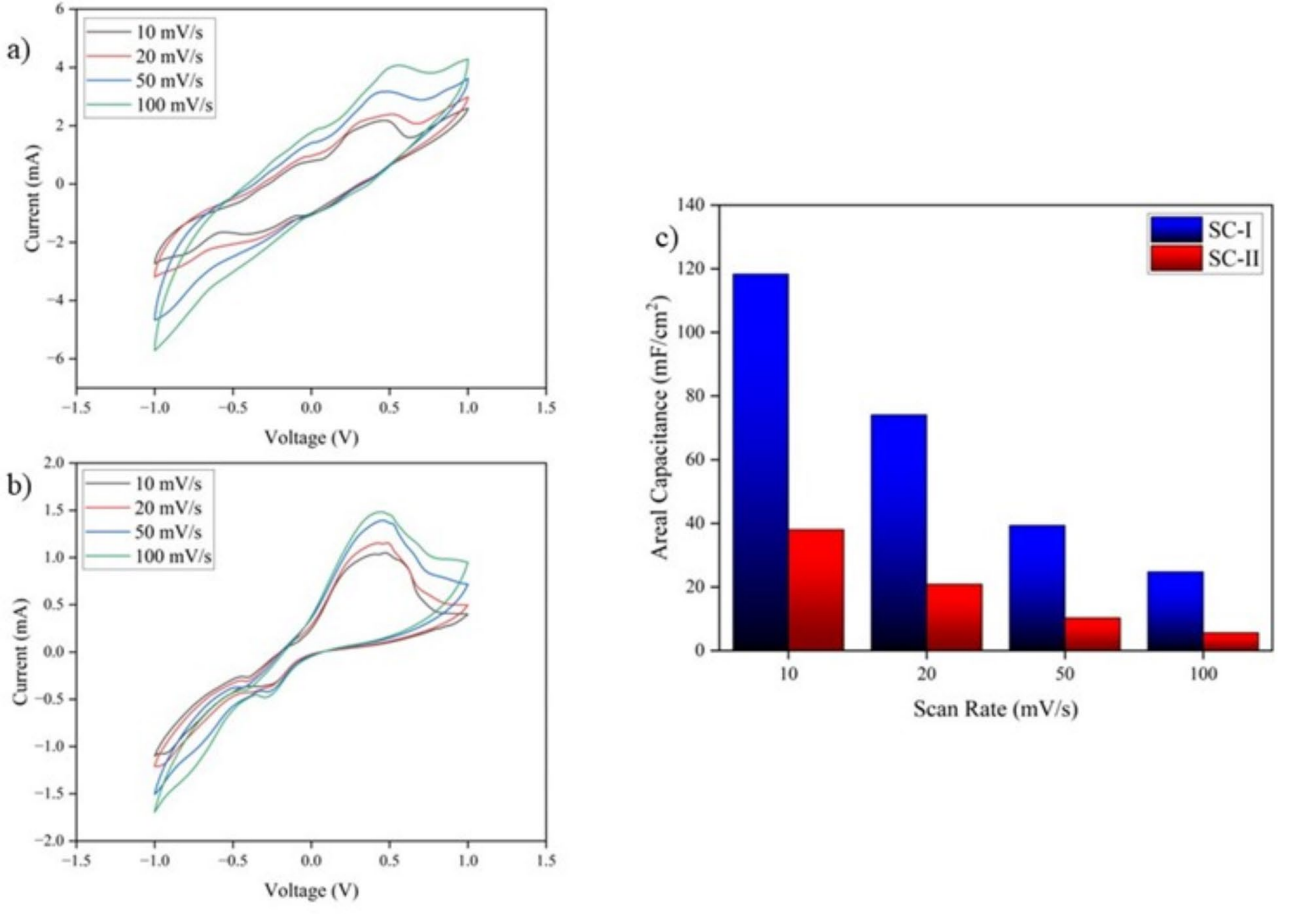
(**a**) CV of SC-I (**b**) CV of SC-II (**c**) Comparison of Areal Capacitance at scan rates 10 mV/s, 20 mV/s, 50 mV/s, 100 mV/s.

CV of SC-I and SC-II were compared to understand the performance metrics between screen-printed and drop coated electrodes. CV was conducted for scan rates 10 mV/s 20 mV/s, 50 mV/s, and 100 mV/s between the voltage window -1 V to 1 V as shown in Fig. 10. Both the supercapacitors exhibited a quasi-reversible process and a significant oxidation peak at 0.47 V.

The CV curves show that the SC-I exhibited high areal capacitance of 118 mF/cm^2^ at 10 mV/s and 25.76 mF/cm^2^ at 100 mV/s. This is significantly higher compared to SC-II which exhibited an areal capacitance of 38.02 mF/cm^2^ at 10 mV/s and 5.63 mF/cm^2^ at 100 mV/s. Drop coating of Ag nanoparticles into the graphene structure of SC-I showed improved pore utilization. This enhancement is attributed to the structural changes induced by the nanoparticles, which likely reduced the Van Der Waal interaction between Graphene sheets leading to higher inter-layer spacing^41^. Pore size plays a very vital role in electrochemical performance. Higher pore size can result in better electrolyte diffusion. As a result, SC-I demonstrated a more efficient utilization of the intrinsic graphene pore structure, providing a highly accessible surface area for ion-penetration and diffusion. This feature promotes faster ion transport, leading to higher current density compared to SC-II as shown ^42^. In this study, the existing graphene macropores are enhanced by anchoring Ag nanoparticles resulting in enhanced pore utilization and electrochemical activity.

In contrast, the screen-printing technique used in SC-II for depositing Ag nanoparticles resulted in less efficient pore utilization. The highly agglomerated non-porous structure hinders electrolyte penetration and ion transport. The reduced surface area causes a reduction in capacitance as well, which is consistent with the SEM images revealing blocked graphene pores.

The results obtained also contradict the sheet resistance offered by the electrodes. This shows that electrode performance is greatly mediated or influenced by pore characteristics. Low sheet resistance might translate to excellent ion-transfer capability, but not areal capacitance.

#### GCD results

**Fig. 11:**
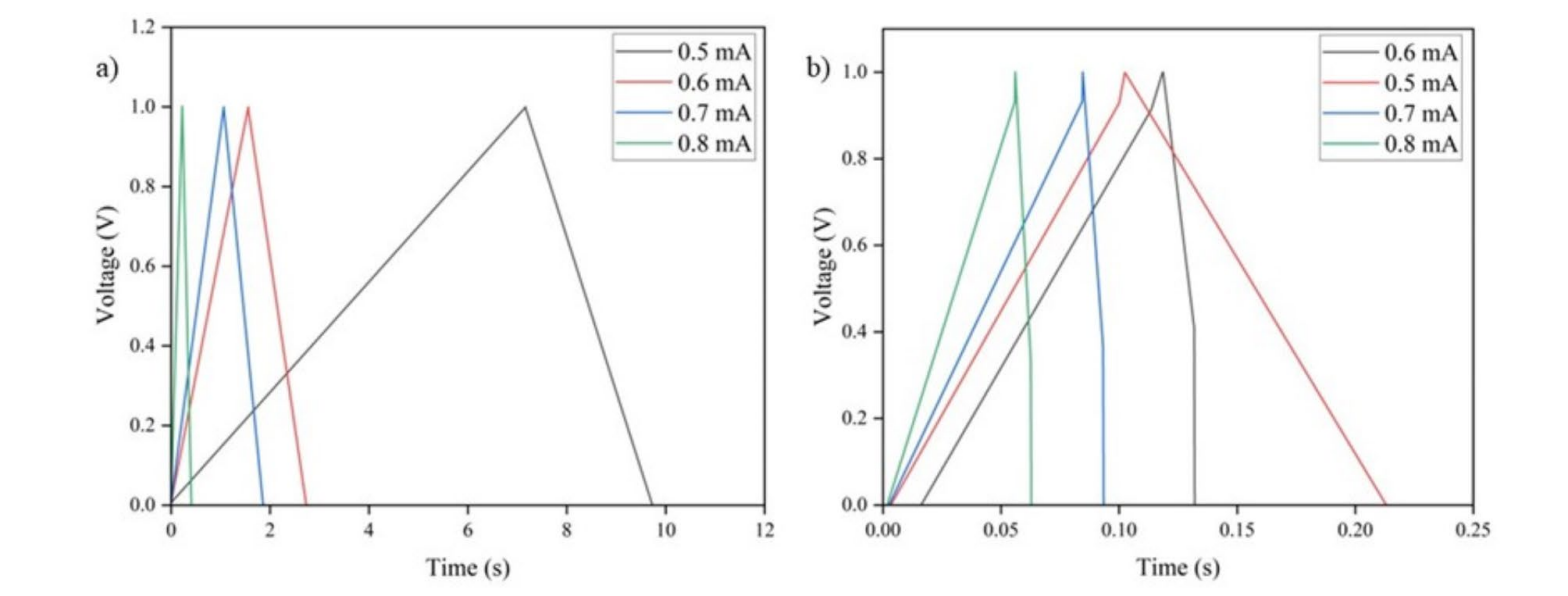
GCD curves of (**a**) SC-I and (**b**) SC-II.

GCD was carried out for 4 different current densities for SC-I and SC-II as shown in Fig. 11 between 0 and 1 V. The asymmetric charge–discharge curve and negligible IR drop is observed, is indicative of superior electrochemical reversibility of SC-I in comparison to SC-II. GCD curves of both SC-I and SC-II have a triangular shape, a characteristic of double layer performance. However, a larger discharge curve is observed for all current densities for SC-I in comparison to SC-II. Additionally, an ESR of 66 Ω resulted in a very small iR_drop_ as shown in Fig. 11(b). This difference can be attributed to the efficient charge storage and electron diffusion owing to the synergistic effects of AgNPs and the Graphene nanostructures.

The areal capacitance values varied were calculated from the GCD curves using Eq. (2). SC-I and SC-II showed a capacitance of 4.5 mF/cm^2^ and 0.1 mF/cm^2^ respectively at 0.5 mA/cm^2^ current density. The energy density and power density were calculated from the following equations:

3 $$E=\frac{{C}_{S}\times {\Delta V}^{2}}{2}$$

4 $$P=\frac{E}{\Delta t}$$

The values of energy density and power density are as shown in Table 4.

**Table 4:** Energy and Power Density of SC-I and SC-II.

Supercapacitor	Energy Density (mWh/cm^2^)	Power Density (mW/cm^2^)
I	2.42	0.25
II	0.05	0.21

#### Cycling and Coulombic Efficiency

**Fig. 12:**
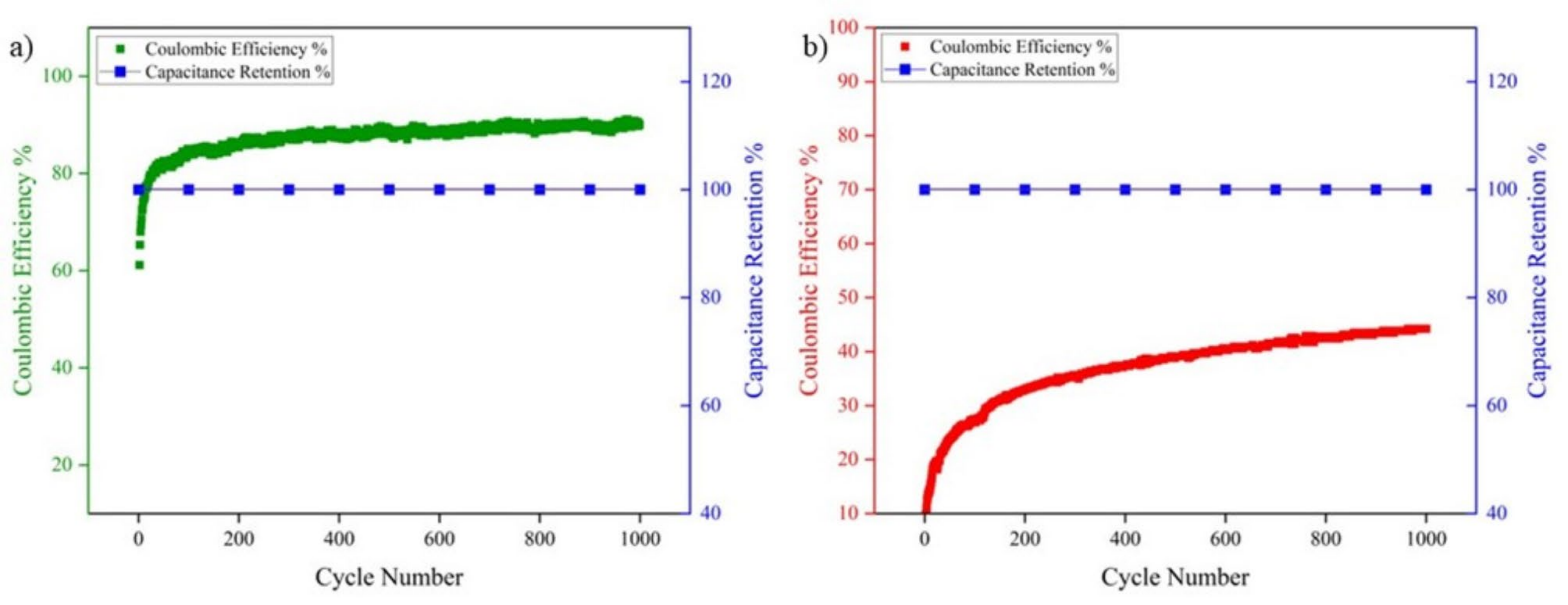
Cycling and Coulombic Efficiency of (**a**) SC-I and (**b**) SC-II.

Cycling stability is an important parameter to study the long-term efficiency of the supercapacitor. As shown in Fig. 12, both Capacitance Retention and Coulombic Efficiency are plotted. The latter is given by the equation:

5 $$\eta \%= \frac{{t}_{d}}{{t}_{c}}\times 100$$

For 1000 cycles, SC-I exhibited an excellent capacitance retention of 100% and a Coulombic Efficiency of 90.1%. On the other hand, SC-II exhibited a 100% capacitance retention but poor Coulombic Efficiency of 44.2%. The synergy of both graphene and AgNPs in SC-I is indicative of the high cycling stability. In the case of SC-II, the low coulombic efficiency can be attributed to irreversible redox reactions at the electrode–electrolyte interface.

#### EIS results

**Fig. 13:**
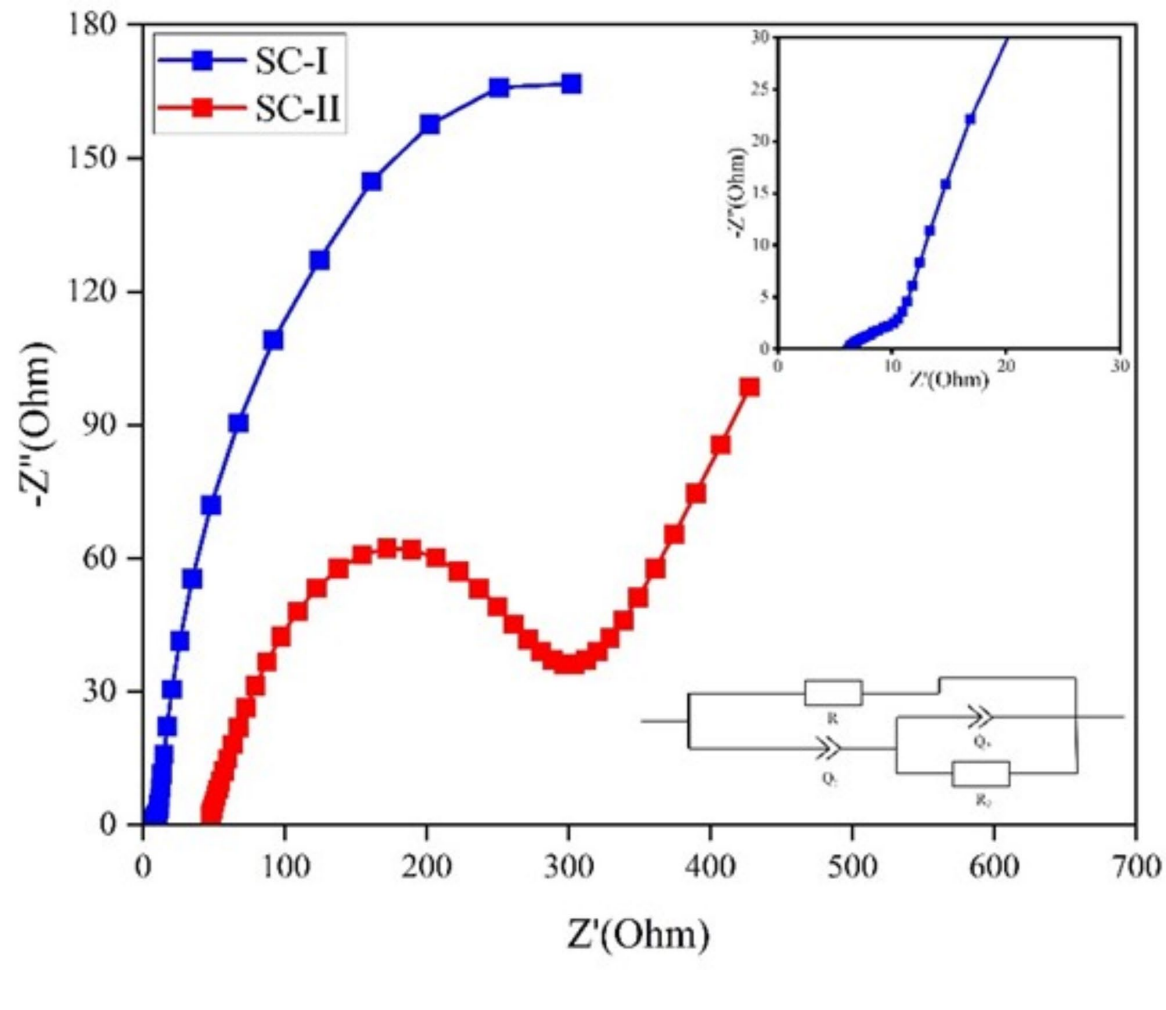
EIS of SC-I and SC-II.

EIS measurements were conducted over a frequency range of 100 mHz to 1 MHz as shown in Fig. 13, to study different resistive parameters such as bulk resistance, charge-transfer resistance, and kinetic processes.

The EIS graph typically consists of both linear and semi-circular regions. The linear portion corresponds to a diffusion-limited process, characteristic of a supercapacitor. The semi-circle in the graph is indicative of the double-layer capacitance, which is a result of the symmetric nature of the supercapacitor. Z-fitting of the EIS data was performed using EC-LAB software to create an equation that best fits the plot. In this equation, R_1_ and R_2_ represent the bulk resistance and charge transfer resistance respectively. Additionally, Q_1_ and Q_2_ represent a "constant phase element" used to accommodate any non-ideal behaviour of the supercapacitor system.

From the graph, it is evident that the Nyquist curves for both composites are linear at low frequencies indicating the capacitive behaviour of the supercapacitors. A larger linear slope in the low frequency region of the Nyquist curve of SC-I in comparison to SC-II represents a faster ion diffusion and a better energy storage capability of the electrode material of SC-I than SC-II ^43,44^. It is evident from the EIS results, that incorporation of AgNP using drop-coating reduce the resistance offered by the supercapacitor (R_1_ = 6.19 Ω; R_2_ = 10.91 Ω) significantly and accelerates charge-transfer and ion-diffusion process within the layers of graphene. This can be attributed to improved surface characteristics due to homogenous distribution of AgNPs ^20^. However, screen-printing does not allow the effective utilization of Graphene layers and hence the high charge transfer and bulk resistance (R_1_ = 25.8 Ω; R_2_ = 300.26 Ω).

**Fig. 14:**
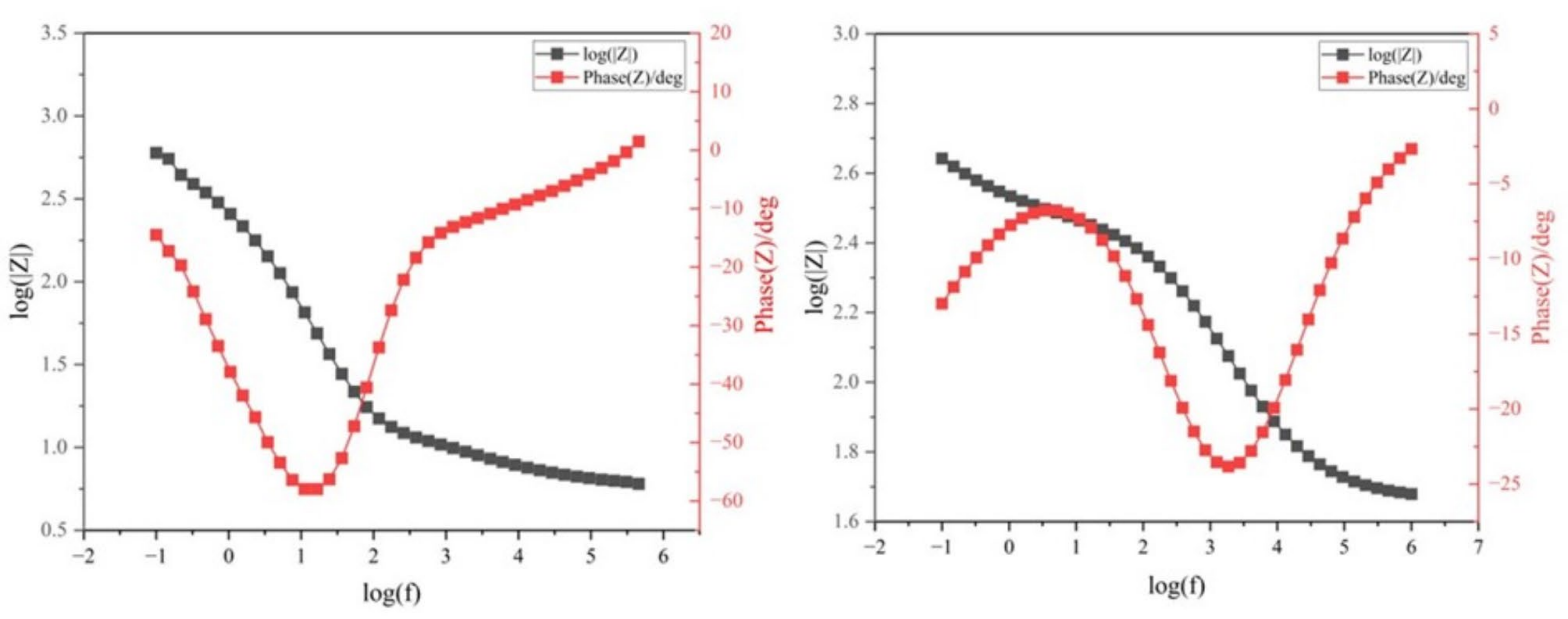
Bode Plot of (**a**) SC-I and (**b**) SC-II.

In addition to the Nyquist plot, a Bode plot (Fig. 14) was plotted to further study the supercapacitor system to study the response time of the supercapacitor with respect to changes in voltage and current. The bode plot is composed of two parts. The first is the Impedance Magnitude vs Frequency the second is Phase angle vs frequency. The former plot provides insights into how the impedance of the supercapacitor varies across different frequencies. The latter provides information about the phase shift between the voltage and current in the supercapacitor. The time constant (*τ*_*o*_*)* can be derived from this plot to study response time of the supercapacitors ^45^. It is the point when the supercapacitor transitions from resistive behaviour at high frequency to capacitive behaviour at low frequencies. It is given as:

6 $${\tau }_{0}= \frac{1}{\text{log}\left({f}_{0}\right)}$$

where ${f}_{0}$ is the frequency at which $\text{log}\left(\left|Z\right|\right)=phase(Z)$. From Eq. (5), the values of ${\tau }_{0}$ are 0.35 s and 0.88 s for SC-I and SC-II respectively. The smaller time constant from SC-I is indicative of a faster charge transfer process which is consistent with the discussion from the Nyquist Plot.

### Antifungal studies

**Fig. 15:**
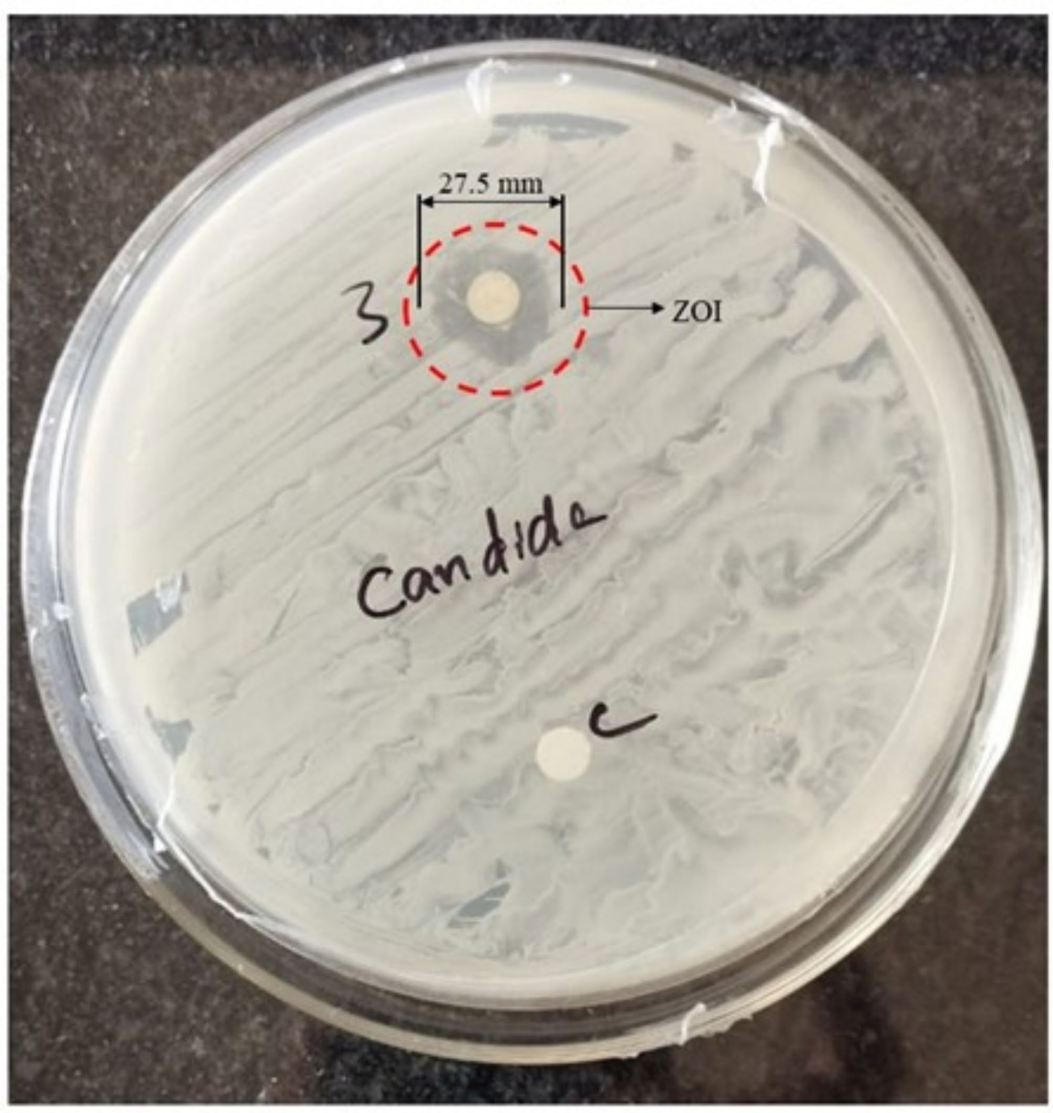
Evaluation of the antifungal activity against Candida sp. by agar well diffusion method.

The antifungal activity of the developed E_2_ composite was tested against *Candida sp*. In this study, the composite exhibited the zone of inhibition (ZOI) of 27.5 mm (as highlighted in red) against the *Candida sp.* after 24 h of incubation at 37 °C (Fig. 15). Our results showed a good antifungal effect against *Candida sp.* This is the first report on the antifungal potential of the developed E_2_ composite.

## Conclusion

Through this work, we studied the differences in performance metrics of a composite prepared using two methods: screen-printing and drop coating. AgNPs prepared using green method have been instrumental in increasing the performance of a Laser-Induced Graphene based supercapacitor by using drop-coating. This composite architecture and method of preparation is more cost effective and easily achieved as opposed to chemical methods of AgNO_3_ reduction. The supercapacitor exhibited an areal capacitance of 118 mF/cm^2^, at 10 mV/s, which is roughly 5 times that of the supercapacitor prepared using highly conducting screen-printed electrodes. The screen-printed electrode exhibited a conductivity of 151.09 S/cm which is roughly 13 times of the electrode conductivity prepared from drop coating. Despite the significant difference in the electrode conductivity using the 4-probe method, it is evident that the capacitance offered by the latter is higher. This proves that areal capacitance greatly depends on the surface and pore characteristics of the electrode. Owing to the existing challenges in fighting fatal fungal infections, we found it imperative to test our composite for its anti-fungal properties against *Candida sp.* The combination of AgNPs with Laser-Induced Graphene using screen-printing proved to be an effective composite with antifungal capabilities.

## Data Availability

The datasets used and/or analyzed during the current study are available from the corresponding author on reasonable request.
